# Physical activity partly mediates the association between cognitive function and depressive symptoms

**DOI:** 10.1038/s41398-022-02191-7

**Published:** 2022-09-27

**Authors:** Zsófia Csajbók, Stefan Sieber, Stéphane Cullati, Pavla Cermakova, Boris Cheval

**Affiliations:** 1 Faculty of Humanities, Charles University, Prague, Czech Republic; 2 LIVES Centre, Swiss Centre of Expertise in Life Course Research, Lausanne, Switzerland; 3 Center for the Interdisciplinary Study of Gerontology and Vulnerability, University of Geneva, Geneva, Switzerland; 4 Population Health Laboratory, University of Fribourg, Fribourg, Switzerland; 5 Department of Readaptation and Geriatrics, University of Geneva, Geneva, Switzerland; 6 Second Faculty of Medicine, Charles University, Prague, Czech Republic; 7 National Institute of Mental Health, Klecany, Czech Republic; 8 Swiss Center for Affective Sciences, University of Geneva, Geneva, Switzerland; 9 Laboratory for the Study of Emotion Elicitation and Expression (E3Lab), Department of Psychology, University of Geneva, Geneva, Switzerland

**Keywords:** Depression, Human behaviour

## Abstract

Cognitive function, physical activity, and depressive symptoms are intertwined in later life. Yet, the nature of the relationship between these three variables is unclear. Here, we aimed to determine which of physical activity or cognitive function mediated this relationship. We used large-scale longitudinal data from 51,191 adults 50 years of age or older (mean: 64.8 years, 54.7% women) from the Survey of Health, Ageing and Retirement in Europe (SHARE). Results of the longitudinal mediation analyses combined with autoregressive cross-lagged panel models showed that the model with physical activity as a mediator better fitted the data than the model with cognitive function as a mediator. Moreover, the mediating effect of physical activity was 8–9% of the total effect of cognitive function on depressive symptoms. Our findings suggest that higher cognitive resources favor the engagement in physical activity, which contributes to reduced depressive symptoms.

## Main

Engaging in regular physical activity and maintaining high cognitive function are essential for health [1–3]. Thus, the age-related decline in physical activity and cognitive function [4–6] often affects mental health [3, 7, 8]. Yet, the nature of the relationship between physical activity, cognitive function, and mental health across aging remains unclear.

Physical activity has been found to reduce the risk of developing depressive symptoms [9–11] through several biological and psychosocial pathways [12, 13]. Likewise, a recent study drawing on large-scale genome-wide association studies revealed that a device-based measure of physical activity reduces the risk of major depression, while the association in the opposite direction was not significant [14]. In addition, longitudinal studies showed that cognitive decline precedes the emergence of depressive symptoms [15–18], while the opposite association was not observed. Thus, previous literature suggests that both physical activity and cognitive function predict subsequent changes in depressive symptoms. However, the temporal precedence and directionality governing the effect of physical activity and cognitive function is unclear. Although observational results suggested that physical activity enhanced cognitive function [19–21], more recent studies showed that higher levels of cognitive function can increase the engagement in physical activity [22–25]. The effect of physical activity on cognitive function can be explained by the effects of physical activity on angiogenesis, neurogenesis, cortical thickness, and growth factor production [26–28]. The effect in the opposite direction (cognitive function → physical activity) can be explained by experimental and theoretical work related to the theory of effort minimization [29–34]. According to this perspective on effort minimization, engaging in physical activity requires cognitive resources to override the automatic attraction toward effort minimization.

**Fig. 1:**
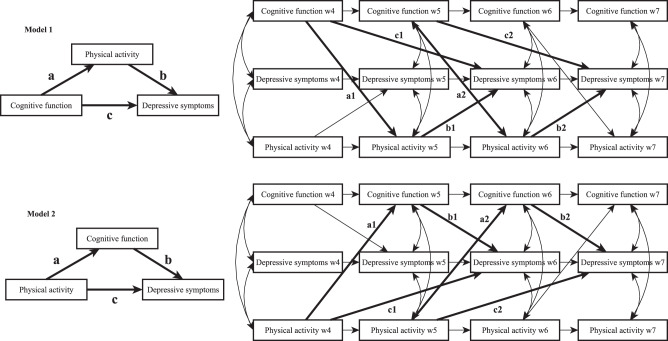
Hypothesized autoregressive longitudinal mediation models. Top panel: Model 1 testing the mediating role of physical activity in the association between cognitive function and depressive symptoms. Bottom panel: Model 2 testing the mediating role of cognitive function in the association between physical activity and depressive symptoms.

Based on the aforementioned literature, two models can be hypothesized (Fig. 1). First, a decline in physical activity has a detrimental effect on cognitive function, which contributes to depressive symptoms. Second, a decline in cognitive function has a detrimental effect on physical activity, which contributes to depressive symptoms. In other words, cognitive function may mediate the effect of physical activity on depressive symptoms (physical activity → cognitive function → depressive symptoms). Alternatively, physical activity may mediate the effect of cognitive function on depressive symptoms (cognitive function → physical activity → depressive symptoms). These hypotheses are not mutually exclusive as a vicious cycle between cognitive decrement and physical inactivity could also occur [23]. The objective of the present study was to test these two hypothesized models. Understanding the nature of the relationship between physical activity, cognitive function, and depressive symptoms in adults 50 years of age or older can contribute to improving interventions aiming to promote mental health in this population.

## Results

We studied 51,191 individuals (55% women, mean age at baseline 65 years). Table 1 summarizes the baseline characteristics of the analyzed sample. Table 2 describes the data of each measure across waves.

**Table 1:** Baseline characteristics of participants (*n* = 51,191).

Age, mean ± SD	64.75 ± 9.14
Women, *n* (%)	27,99 (54.7)
Moderate physical activity, *n* (%)	
More than once a week	37,60 (73.5)
Once a week	7,13 (13.9)
One to three times a month	2,77 (5.4)
Hardly ever or never	3,69 (7.2)
Vigorous physical activity, *n* (%)	
More than once a week	19,55 (38.1)
Once a week	7,96 (15.6)
One to three times a month	5,05 (9.9)
Hardly ever or never	18,67 (36.5)
Cognitive function	
Delayed recall, mean ± SD	4.05 ± 2.09
Verbal fluency, mean ± SD	20.81 ± 7.55
Depressive symptoms, median (IQR)	2.00 (2.00)

*SD* standard deviation, *IQR* interquartile range.

**Table 2:** Descriptive statistics of study measures at each wave.

		Wave 4	Wave 5	Wave 6	Wave 7
Delayed recall	*N*	35215	45323	43411	36606
	*M* (SD)	4.069 (2.108)	4.145 (2.154)	4.106 (2.173)	3.95 (2.154)
Depressive symptoms	*N*	35125	45369	43405	11511
	*M* (SD)	2.005 (1.700)	2.021 (1.902)	2.112 (2.000)	2.165 (2.097)
Moderate physical activity	*N*	35331	45603	43928	11734
	*M* (SD)	3.527 (0.901)	3.525 (0.912)	3.500 (0.943)	3.415 (1.013)
Vigorous physical activity	*N*	35328	45596	43926	11731
	*M* (SD)	2.522 (1.324)	2.531 (1.329)	2.452 (1.327)	2.315 (1.299)
Verbal fluency (divided by 10)	*N*	35196	45407	43458	11562
	*M* (SD)	2.089 (0.751)	2.137 (0.746)	2.097 (0.779)	1.927 (0.81)

*M* mean, *SD* standard deviation.

### Longitudinal mediation analyses

Table 3 presents the results of the longitudinal mediation models. In Model 1 (cognitive function → physical activity → depressive symptoms), results showed that higher cognitive function predicted higher physical activity 2 years later (a1: *B* = 0.022, *p* < 0.001; a2: *B* = 0.030, *p* < 0.001) and that higher physical activity predicted lower depressive symptoms after 2 more years (b1: *B* = −0.137, *p* < 0.001; b2: −0.137, *p* < 0.001). Results further demonstrated an indirect effect of cognitive function on depressive symptoms through physical activity (indirect1: *B* = −0.003, *p* < 0.001; indirect2: *B* = −0.004, *p* < 0.001). After adjusting for physical activity, results showed that lower cognitive function predicted higher depressive symptoms 4 years later (c’1: *B* = −0.036, *p* < 0.001; c’2: *B* = −0.040, *p* < 0.001), which suggested that the effect of cognitive function on depressive symptoms was not fully mediated by physical activity. Specifically, the proportion of the total effect that was mediated by physical activity was 8% between waves 4 and 6, and 9% between waves 5 and 7.

**Table 3:** Longitudinal mediation models (*n* = 51,191).

	Model 1 Cognitive function → (a) physical activity → (b) depressive symptoms	Model 2 Physical activity → (a) cognitive function → (b) depressive symptoms
	Unstandardized B (95% CI)	Unstandardized B (95% CI)
a1	0.022*** (0.018, 0.027)	0.108*** (0.086, 0.130)
b1	−0.137*** (−0.159, −0.115)	−0.038*** (−0.046, −0.029)
c’1 (Direct effect)	−0.036*** (−0.046, −0.027)	−0.119*** (−0.145, −0.094)
Total effect 1	−0.039*** (−0.049, −0.030)	−0.123*** (−0.149, −0.099)
Indirect effect 1	−0.003*** (−0.004, −0.002)	−0.004*** (−0.005, −0.003)
Proportion of mediation effect 1	8%	3%
a2	0.030*** (0.026, 0.034)	0.101*** (0.081, 0.121)
b2	−0.137*** (−0.178, −0.096)	−0.048*** (−0.065, −0.031)
c’2 (Direct effect)	−0.040*** (−0.056, −0.021)	−0.101*** (−0.150, −0.051)
Total effect 2	−0.044*** (−0.063, −0.025)	−0.106*** (−0.155, −0.056)
Indirect effect 2	−0.004*** (−0.006, −0.003)	−0.005*** (−0.007, −0.003)
Proportion of mediation effect 2	9%	5%
AIC	1527866.912	1527970.946
*X*^2^(df)	9758.894 (37)***	9862.928 (37)***
RMSEA (95% CI)	0.072 (0.070,0.073)	0.072 (0.071,0.073)
CFI	0.909	0.908
TLI	0.750	0.747
SRMR	0.059	0.060

The analysis was performed using cross-lagged panel longitudinal mediation model. The observed depressive symptoms, physical activity, and cognitive function variables were regressed on sex, age, and sex × age in each wave. The “1” describes the associations between wave 4 and wave 6, while the “2” describes the associations between wave 5 and wave 7, see Fig. 1.

*AIC* Akaike Information Criterion, *CFI* comparative fit index, *CI* confidence interval, *RMSEA* root-mean square error of approximation, *SRMR* standardized root-mean-squared residual, *TLI* Tucker-Lewis index.

***p < 0.001.

In Model 2 (physical activity → cognitive function → depressive symptoms), results showed that higher physical activity predicted higher cognitive function 2 years later (a1: *B* = 0.108, *p* < 0.001; a2: *B* = 0.101, *p* < 0.001) and higher cognitive function predicted lower depressive symptoms after 2 more years (b1: *B* = −0.038, *p* < 0.001; b2: *B* = −0.048, *p* < 0.001). Results further demonstrated a significant indirect effect of physical activity on depressive symptoms through cognitive function (indirect1: *B* = −0.004, *p* < 0.001; indirect2: *B* = −0.005, *p* < 0.001). After adjusting for cognitive function, results showed that lower physical activity significantly predicted higher depressive symptoms 4 years later (c’1: *B* = −0.119, *p* < 0.001; c’2: *B* = −0.101, *p* < 0.001), which suggested that the effect of physical activity on depressive symptoms was not fully mediated by cognitive function. Specifically, the proportion of the total effect that was mediated by cognitive function was 3% between waves 4 and 6, and 5% between waves 5 and 7.

Overall, results showed that the model including physical activity as a mediator of the association between cognitive function and depressive symptoms (Model 1) fitted the data more accurately than the model including cognitive function as a mediator of the association between physical activity and depressive symptoms (Model 2) when compared with Akaike Information Criterion (AIC): AIC_Model1_ – AIC_Model2_ = −104.034 (Table 3). This finding was consistent with the observation that the proportion of the total effect mediated by physical activity was about two times larger than the one mediated by cognitive function (8% vs. 3%, and 9% vs. 5%, respectively; Table 3).

### Sensitivity analyses

Results of the sensitivity analyses were comparable with those of the main analyses for both Models 1 and 2 (Supplementary Table 1). Specifically, in Model 1, when verbal fluency was used instead of delayed recall, results showed an indirect effect of cognitive function on depressive symptoms through physical activity with a comparable proportion of mediated effect (i.e., 8 and 11%). When vigorous physical activity replaced moderate physical activity, the mediating pattern was similar, but the proportion of mediated effect was smaller (i.e., 5 and 7%). In Model 2, the proportion of mediated effects of 3 and 5% were observed, when verbal fluency replaced delayed recall, or 4 and 4%, when vigorous physical activity replaced moderate physical activity. AIC_Model1_ – AIC_Model2_ = −620.719 when verbal fluency replaced delayed recall, but AIC_Model1_ – AIC_Model2_ = 50.983 when vigorous activity replaced moderate activity.

### The moderating effect of sex and age

As the interaction term of sex × age was a significant predictor of cognitive function, physical activity, and depressive symptoms, we replicated the cross-lagged panel models (CLPM) across sex and age categories. The results were comparable to the main models in the direction of the effects (Table 4). In Model 1, women above the age of 65 experienced the highest total effect of cognitive function on depressive symptoms (total effect1: *B* = −0.069, *p* < 0.001; total effect2: *B* = −0.087, *p* < 0.001), but the proportion of mediation was the highest in women younger than 65 years in the first mediation cycle (17%) with the second lowest total effect (total effect1: *B* = −0.024, *p* < 0.05). The proportion of mediation was the lowest in men younger than 65 years (effect1: 3%). Moreover, like in the main analysis, the proportion of mediating effect was smaller in Model 2 (2–9%) than in Model 1 (3–17%). In Model 2, women younger than 65 showed the highest total effect of physical activity on depressive symptoms (total effect1: *B* = −0.186, *p* < 0.001), but with only a 2% mediating effect. The highest proportion of mediating effect was observed in men and women older than 65 years (both 9%).

**Table 4:** Longitudinal mediation models across sex and age.

	Model 1 Cognitive function → (a) physical activity → (b) depressive symptoms				Model 2 Physical activity → (a) cognitive function → (b) depressive symptoms			
	Men		Women		Men		Women	
	<65	≥65	<65	≥65	<65	≥65	<65	≥65
*n*	12,109	11,083	15,335	12,664	12,109	11,083	15,335	12,664
	Unstandardized B (95% CI)				Unstandardized B (95% CI)			
a1	0.007 (−0.002, 0.016)	0.029*** (0.019, 0.039)	0.022*** (0.014, 0.030)	0.057*** (0.047, 0.066)	0.054* (0.003, 0.105)	0.120*** (0.077, 0.165)	0.113*** (0.065, 0.159)	0.176*** (0.138, 0.214)
b1	−0.097*** (−0.143, −0.052)	−0.151*** (−0.195, −0.107)	−0.176*** (−0.222, −0.129)	−0.131*** (−0.172, −0.090)	−0.028** (−0.045, −0.012)	−0.057*** (−0.076, −0.039)	−0.023** (−0.039, −0.007)	−0.067*** (−0.085, −0.050)
c’1 (Direct effect)	−0.030** (−0.049, −0.011)	−0.059*** (−0.079, −0.039)	−0.021* (−0.040, −0.002)	−0.062*** (−0.082, −0.041)	−0.063* (−0.114, −0.011)	−0.102*** (−0.152, −0.051)	−0.183*** (−0.236, −0.131)	−0.120*** (−0.167, −0.073)
Total effect 1	−0.030** (−0.049, −0.011)	−0.064*** (−0.084, −0.043)	−0.024* (−0.043, −0.006)	−0.069*** (−0.089, −0.049)	−0.064* (−0.115, −0.013)	−0.108*** (−0.158, −0.058)	−0.186*** (−0.238, −0.133)	−0.132*** (−0.179, −0.085)
Indirect effect 1	−0.001 (−0.002, 0.000)	−0.004*** (−0.007, −0.003)	−0.004*** (−0.006, −0.002)	−0.007*** (−0.010, −0.005)	−0.002 (−0.004, 0.000)	−0.007*** (−0.011, −0.004)	−0.003* (−0.005, −0.001)	−0.012*** (−0.016, −0.008)
Proportion of mediation effect 1	3%	6%	17%	10%	3%	6%	2%	9%
a2	0.013** (0.016, 0.050)	0.045*** (0.035, 0.056)	0.030*** (0.023, 0.037)	0.055*** (0.045, 0.064)	0.067** (0.020, 0.113)	0.097*** (0.057, 0.136)	0.129*** (0.089, 0.170)	0.162*** (0.127, 0.198)
b2	−0.083 (−0.169, 0.004)	−0.176*** (−0.255, −0.098)	−0.104* (−0.197, −0.011)	−0.164*** (−0.237, −0.093)	−0.011 (−0.045, 0.023)	−0.107*** (−0.141, −0.073)	−0.021 (−0.053, 0.012)	−0.074*** (−0.106, −0.041)
c’2 (Direct effect)	0.017 (−0.021, 0.054)	−0.072*** (−0.109, −0.035)	−0.032 (−0.068, 0.004)	−0.078*** (−0.114, −0.041)	−0.053 (−0.162, 0.057)	−0.096* (−0.188, −0.007)	−0.070 (−0.175, 0.032)	−0.163*** (−0.249, −0.079)
Total effect 2	0.016 (−0.022, 0.053)	−0.080*** (−0.117, −0.043)	−0.035 (−0.070, 0.001)	−0.087*** (−0.123, −0.050)	−0.054 (−0.162, 0.056)	−0.106* (−0.198, −0.017)	−0.072 (−0.177, 0.029)	−0.175*** (−0.261, −0.091)
Indirect effect 2	−0.001 (−0.003, 0.000)	−0.008*** (−0.012, −0.004)	−0.003* (−0.006, 0.000)	−0.009*** (−0.013, −0.005)	−0.001 (−0.004, 0.002)	−0.010*** (−0.016, −0.005)	−0.003 (−0.007, 0.002)	−0.012*** (−0.018, −0.006)
Proportion of mediation effect 2	NA	10%	9%	10%	2%	9%	4%	7%
AIC	339384.279	330806.444	452378.299	405070.761	339400.979	330891.292	452360.175	405187.988
*X*^2^ (df)	1779.333 (37)***	2391.406 (37)***	3074.899 (37)***	3234.749 (37)***	1796.033 (37)***	2476.254 (37)***	3056.775 (37)***	3351.976 (37)***
RMSEA (95% CI)	0.062 (0.060–0.065)	0.076 (0.073–0.078)	0.073 (0.071–0.075)	0.083 (0.080–0.085)	0.063 (0.060–0.065)	0.077 (0.075–0.080)	0.073 (0.071–0.075)	0.084 (0.082–0.087)
CFI	0.880	0.867	0.859	0.870	0.879	0.863	0.860	0.865
TLI	0.796	0.774	0.759	0.779	0.795	0.766	0.761	0.770
SRMR	0.065	0.086	0.074	0.089	0.066	0.092	0.075	0.096

The analysis was performed using cross-lagged panel longitudinal mediation model. *NA* not applicable, the proportion of mediating effect is not calculated, because the signs of the indirect and total effects are different. The “1” describes the associations between wave 4 and wave 6, while the “2” describes the associations between wave 5 and wave 7, see Fig. 1.

*AIC* Akaike Information Criterion, *CFI* comparative fit index, *CI* confidence interval, *RMSEA* root-mean square error of approximation, *SRMR* standardized root-mean-squared residual, *TLI* Tucker-Lewis index.

*p < 0.05. **p < 0.01. ***p < 0.001.

### Complementary analyses

In the random intercepts cross-lagged panel model (RI-CLPM), history-independent autoregressive latent trajectory model (HI-ALT), and autoregressive latent trajectory model with structured residuals (ALT-SR) approaches, the mediating effects of physical activity in Model 1 and of cognitive function in Model 2 were ~0% (Supplementary Table 1). The results of the fully saturated CLPM model were comparable with the CLPM model in that Model 1 had higher proportion of mediating effects (2–4%) than Model 2 (1–2%; Supplementary Table 1). Finally, the XM interaction models obtained small and non-significant estimates of the exposure-mediator interaction.

## Discussion

### Main findings

Based on a sample of 51,191 adults aged 50 years or older used to investigate the relationship between physical activity, cognitive function, and depressive symptoms, our results showed the model with physical activity as a mediator better fitted the data than the model with cognitive function as a mediator. Moreover, the mediating effect of physical activity was 8–9% of the total effect of cognitive function on depressive symptoms, whereas the mediating role of cognitive function was 3–5% of the total effect of physical activity on depressive symptoms. Finally, age and sex moderated the effects observed, with the highest proportion of mediated effect observed in younger women (i.e., 17%), and the lowest proportion observed in younger men (i.e., 3%). Altogether, these findings suggest that higher cognitive resources favor the engagement in physical activity, which contributes to reduced depressive symptoms.

### Comparison with other studies

Our results showed that higher engagement in physical activity predicts lower depressive symptoms, which is consistent with the literature that have robustly demonstrated a protective role of physical activity on mental health [9, 10]. Our findings confirm this association and add to the mounting evidence showing that physical activity prospectively predicts the level of depressive symptoms. Several psychosocial and biological scenarios have been put forth to explain this protective role [26, 28, 35]. Likewise, our results showing that higher levels of cognitive function predicted lower depressive symptoms were consistent with previous evidence indicating that cognitive decline precedes depressive symptoms in later life [15–18]. Several mechanisms including the detrimental effect of cognitive decline on the ability to be independent in daily life activities [36] and the awareness of their own cognitive decline [37] can lead to an increase in depressive symptoms. In addition, a common neurodegenerative process and cerebrovascular diseases have been proposed to explain the age-related decline in cognitive function and the increase in depressive symptoms [38].

To the best of our knowledge, our large-scale longitudinal study is the first one to investigate the mediation mechanisms that underlie the relationship between cognitive function, physical activity, and depressive symptoms in adults 50 years of age or older. We found that physical activity partly mediated the effect of cognitive function on depressive symptoms, while the mediating role of cognitive function in the association between physical activity and depressive symptoms was less convincing. These findings suggest that the age-related cognitive decline precedes the decline in physical activity, which is consistent with the literature demonstrating that cognitive resources are required to engage in physical activity [22–25]. One plausible scenario for this observation can be found in the theory of effort minimization in physical activity (TEMPA) [39, 40]. Specifically, anchored in an evolutionary perspective on physical activity [40, 41], TEMPA argues that individuals hold an automatic tendency for effort minimization that may explain the difficulty to engage in regular physical activity [39]—a proposition that has been confirmed by a large number of studies [29, 31–34, 42, 43]. Crucially, because of such automatic attraction to physical inactivity, TEMPA proposes that cognitive function is essential to counteract this attraction and thereby favor physical activity engagement. Altogether, though not directly assessed, the current findings fit well with TEMPA. It is worth noting that the aforementioned scenarios are not mutually exclusive, as several studies not only demonstrated the protective effect of physical activity on cognitive function [19–21], but also provided biological explanations for this effect [26–28].

Finally, the complementary analyses showed mixed, small, and non-significant within-person effects. This result suggests that within-person changes in cognitive function and physical activity did not predict within-person changes in depressive symptoms. This result can be explained at least in two ways. First, only the between-person approach (main analyses) can model the prospective effect from the start to the end of the follow-up period, while the within-person approach only focuses on the occasion-specific changes in the tested constructs (i.e., one path between two waves). Therefore, each within-person model only tests the respective temporary deviation that applies to that event and does not accumulate the prospective effect throughout the study period [44]. This may suggest that when examining processes that are deteriorating across aging (i.e., biological aging or senescence), statistical approaches allowing to account for the effects that accumulate over time could be more adapted than those focusing on relatively short-term changes. Second, it should be also acknowledged that the lack of a significant association between individual changes in cognitive function and physical activity with depressive symptoms may also reflect that the between-person approaches can be affected by a non-accounted background measure. Yet, previous literature robustly showed that both physical activity and cognitive function predict changes in depressive symptoms, which allows to be rather confident in the veracity of the observed results.

Note that the CLPM models had worse model fit than the other three models in these complementary analyses. However, because CLPM often fits worse than the other models with structured residuals, especially with high sample size, this thus should not be the basis of ruling out the results of the CLPM model [44–46]. Mainly, low CFI and TLI can be attributed to the generally rather weak correlations between the measures (e.g., between depression and both cognitive function measures the correlations are between −0.072 and −0.211 across all waves; between depression and both physical activity measures the correlations are between −0.107 and −0.252; between physical activity and cognitive function the correlations are between 0.110 and 0.252). The model fit could be improved probably by latent variable modeling instead of estimating the CLPM model on observed variables. In the current data, however, there is no fitting, longitudinally invariant measurement model to indicate latent factors, as the two items measuring physical activity and cognitive function were not sufficient to indicate fitting latent factor measurement models. However, the estimated single-level random-effects models obtained practically zero variance of the random effects (all < 0.001), while the XM interaction models obtained small and non-significant estimates of the exposure-mediator interaction, indicating that the basic CLPM and the fully saturated CLPM models are eligible on our data without adjusting for these confounding effects. We also fitted Latent Growth Curve Models (LGCM) against the data to inspect the growth trajectories of the measures independently. Although the slopes were relatively small (the estimated mean latent slope factors were between −0.014 to 0.114 and between −0.058 to 0.003 for the quadratic factors), the significant variances of the latent intercept, slope, and quadratic factors in all measures (except for fluency where only the intercept’s variance was significant) suggested that modeling for the latent growth factors could be a meaningful improvement over the CLPM model. This would support the HI-ALT or the ALT-SR models over the CLPM model, but with two caveats. First, only the CLPM model can demonstrate between-person associations, while the HI-ALT and ALT-SR models focus on within-person associations. Second, the interpretation of the autoregressive latent trajectory models is less straightforward than the simpler models [45]. Still, relying on the LGCM results, if one is interested in the within-person longitudinal mediation effects, the indirect effect is practically zero in both tested models (i.e., Model 1 and 2). Replication studies, including experimental ones, along with the rapidly developing analytical tools, are needed to provide more robust evidence that would help us disentangle the associations between cognitive function, depressive symptoms, and physical activity.

### Strengths and weaknesses

Our large-scale longitudinal study has several strengths. The large sample size from multiple European countries allows a stronger generalization of the current findings compared to studies with smaller and non-international samples. Likewise, the use of longitudinal data has enabled us to assess longitudinal mediation respecting the time-lags and the temporal order between the studied factors. Though correlational, and thereby preventing to definitely probe causal inference, this longitudinal approach still allows to get closer to a test of the causal relationships between physical activity, cognitive function, and depressive symptoms.

However, our study also has some limitations. First and foremost, the physical activity measure was self-reported. Although widely used in previous SHARE-based studies [47–50], self-reported physical activity is prone to inaccuracy and social-desirability biases, which reduces its validity relative to device-based measures [51]. Similarly, studies have observed that the association between physical activity, cognitive function, and depressive symptoms may differ depending on whether physical activity was self-reported or objectively measured [14, 52]. Thus, future studies using a device-based measure of physical activity need to be conducted to test the replicability of the current findings. Second, cognitive function includes various cognitive domains, such as reasoning, processing speed, memory, and spatial ability [53, 54]. Our study relied on delayed recall and verbal fluency, which are thought to reflect memory performance [55] and executive functions [56], respectively. Accordingly, because the associations between physical activity and cognitive functions are likely to depend on the specific cognitive domains assessed, future studies should include additional domains of cognition. Third, the 2-year timespan between the measures was not based on relevant theories of time and change, but because of the features of data collection in SHARE. Accordingly, because the features of the associations between our variables are certainly time sensitive, both in terms of the time difference between measurements (i.e., frequency) and in terms of evolution over the long run (i.e., duration), investigating how the relations observed may have depended on the time frame used is warranted in future studies.

## Conclusion

Our findings show that physical activity partly mediates the effect of cognitive function on depressive symptoms in adults 50 years of age or older. In other words, lower cognitive function may reduce the engagement in physical activity that in turn can elicit higher depressive symptoms. Importantly, only one tenth of the total effect of cognitive function on depressive symptoms was explained by physical activity, which suggests that cognitive function and physical activity have independent effects on depressive symptoms. These findings highlight the need for developing interventions that promote physical activity in cognitively declining adults to limit the onset of depressive symptoms.

## Methods

### Participants and study design

We studied individuals who took part in the Survey of Health, Ageing and Retirement in Europe (SHARE). SHARE is a population-based study of health, social network and economic conditions of community-dwelling individuals, as described in detail elsewhere [57]. The study was initiated in 2004 and assessments have been performed in approximate 2-year intervals. Eligible participants were people 50 years of age or older and their partners, irrespective of age, and were sampled based on probability selection methods. Computer-assisted personal interviewing (CAPI) was used to collect the data in participants´ homes. This study was carried out in accordance with the Declaration of Helsinki. SHARE has been approved by the Ethics Committee of the University of Mannheim (waves 1–4) and the Ethics Council of the Max Plank Society (waves 4–7). All participants provided a written informed consent. Data was pseudo-anonymized, and all participants were informed about the storage and use of the data and their right to withdraw consent.

We restricted the sample to individuals who participated in at least two waves from wave 4 to wave 7 due to the fact that wave 3 (SHARELIFE) did not include the measurements of interest to our study as this wave was devoted to data collection related to childhood histories. Including waves 1 and 2 would have imbalanced the time gaps between measurements across the waves, thereby complicating the estimations of the paths and the comparison of the paths across the different time gaps. Using waves 4 to 7 allowed estimating two complete longitudinal mediation cycles with equal time difference between the measures. Based on these criteria, 76,293 participants were included. Then, we sequentially excluded adults who had less than two measures of depressive symptoms (*n* = 14,738), who had less than two measures of cognitive function (*n* = 801), who had less than two measures of physical activity (*n* = 85), and who were younger than 50 years (*n* = 1300). Finally, we excluded adults with clinically significant depressive symptoms (7 and more points on the EURO-D scale; *n* = 3605) [58, 59], adults who self-reported a diagnosis of dementia, Alzheimer´s disease or senility (*n* = 290), and adults with limitations in activities of daily living (*n* = 4283). The final analytical sample included 51,191 individuals. This sample size was sufficient to perform path-modeling with 37 degrees of freedom [60].

### Measures

#### Physical activity

Physical activity was assessed using the following question: *How often do you engage in activities that require a low or moderate level of energy such as gardening, cleaning the car, or doing a walk?* [50, 61]. Participants answered using a 4-point scale: 4 = more than once a week; 3 = once a week; 2 = one to three times a month; 1 = hardly ever or never. Although this measure cannot be used to accurately determining the prevalence of individuals meeting (or not) the recommended level of physical activity, it has been found to predict a wide range of physical and mental health variables [11, 22, 25, 62].

#### Cognitive function

Cognitive function was assessed with the validated test of delayed recall, which is regarded as a sensitive predictive measure of the development of dementia [55, 63]. Delayed recall was extracted from an adapted 10-word delayed recall test [64]. First, participants listened to a list of 10 words that were read out loud by the interviewer. Then, they were immediately asked to recall as many words as possible. At the end of the cognitive testing session, the participants were asked to recall any of the words from the list a second time, which captured delayed recall. Delayed recall ranged from 0 to 10, with higher scores indicating better cognitive performance. Delayed recall has been shown to be linked to both physical activity and depressive symptoms [15, 25, 65], which makes it a relevant measure for our study. Note, however, that additional measures of cognitive functions, especially those targeting fluid intelligence are needed [15].

#### Depressive symptoms

Depressive symptoms were assessed with the EURO-D scale. The EURO-D scale was originally developed to compare symptoms of late-life depression across 11 European countries in the EURODEP Concerted Action Programme [59] and has been used in many epidemiological studies [16, 66–69]. The 12 items (depressed mood, pessimism, wishing death, guilt, sleep, interest, irritability, appetite, fatigue, concentration, enjoyment, and tearfulness) were scored 0 (symptom not present) or 1 (symptom present), generating a score with a maximum of 12, with higher score indicating more severe depressive symptoms.

### Statistical analyses

We applied a longitudinal mediation analysis to test the two hypothesized models (Fig. 1). The advantage of the longitudinal mediation analysis over the cross-sectional mediation analysis is that the exogenous variable (e.g., cognitive function) precedes the mediator variable (e.g., physical activity) and the outcome variable (i.e., depressive symptoms). Therefore, the longitudinal mediation model accounts for the time-lags and the temporal order that is necessary for testing causal inference [70]. Here, we combined the longitudinal mediation analysis with the CLPM, which is regarded as the best method to test between-person effects [44]. Specifically, the first model (Model 1) longitudinally examined the mediating role of physical activity in the association between cognitive function and depressive symptoms, while adjusting for the autoregressive effects (Fig. 1). Subsequently, Model 2 was tested by creating a similar autoregressive longitudinal mediation model, but with cognitive function as the mediator between physical activity and depressive symptoms (Fig. 1).

The mediating paths were defined longitudinally. Cognitive function at time x predicted physical activity at time *x* + 1 (*path a*) and depressive symptoms at time *x* + 2 (*path c*). Then, physical activity at time *x* + 1 predicted depressive symptoms at time *x* + 2 (*path b*). The respective *a, b*, and *c paths* within the two longitudinal mediations during the four-wave follow-up were freely estimated (i.e., not assuming stationarity) because the models with no equality constraints obtained better AIC than the models assuming stationarity [70]. To estimate the full hypothesized model, depressive symptoms at wave 5 were regressed on physical activity at wave 4, and physical activity at wave 7 was regressed on cognitive function at wave 6. The autoregressive regression paths leading from time *x* to time *x* + 1 within each construct were freely estimated. Similarly, correlation coefficients at each time were freely estimated between the measures obtained at the same time. Maximum Likelihood estimator was used with 10,000 bootstrap replications to estimate asymmetric confidence intervals for the indirect effects and with Full Information Maximum Likelihood estimation for the missing values [71]. Model fit indices were considered to be acceptable if root-mean square error of approximation (RMSEA) and standardized root-mean-squared residual (SRMR) were lower than 0.08, and comparative fit index (CFI) and Tucker-Lewis index (TLI) were higher than 0.9 [72]. The fit of Model 1 and Model 2 were compared based on AIC. The analyses were performed with Mplus 8.7. Mplus syntaxes are in the supplement.

### Moderation analyses

As both age and sex may influence the pattern of results observed [5, 16], the observed variables were regressed on sex, age, and sex × age interaction, thereby allowing to assess potential differences between women and men regarding the evolution of the aforementioned variables across age. The sex × age interaction term was a significant predictor in each model. Each model was therefore re-run stratified by sex (i.e., men vs. women) and two age categories (i.e., <65 vs. ≥65 years) [73].

### Sensitivity analyses

We performed two sensitivity analyses for each model. In the first sensitivity analysis, we used a measure of physical activity of vigorous intensity, derived from the following question: *How often do you engage in vigorous physical activity, such as sports, heavy housework, or a job that involves physical labor?* Participants answered using a 4-point scale. In the second sensitivity analysis, we used a measure of verbal fluency instead of delayed recall. Verbal fluency was derived from the verbal fluency test [74], in which participants were asked to name as many different animals as they could think of within 1 min. The score was the total number of correctly named animals, with a higher score indicating better verbal fluency. For helping model convergence, verbal fluency was divided by 10 to keep the score closer in absolute range to physical activity and depression.

### Complementary analyses

To test the robustness of the CLPM approach, we conducted three complementary analyses relying on different methods. Indeed, the cross-lagged effects can be tested in various ways, with each method allowing specific interpretations about the research question [44]. The most important distinction between cross-lagged models is that they test either for between- or within-person effects. For example, the between-person approach examines whether people with lower cognitive function have a higher risk for lower physical activity and for a greater number of depressive symptoms. In contrast, the within-person approach examines whether, for a particular individual, exhibiting lower cognitive function than usual is associated with a higher risk of physical inactivity and a greater number of depressive symptoms. Here, CLPM was used to test the between-person effects and complementary analyses were used to test the within-person effects or other effects controlled.

In the first complementary analysis, the RI-CLPM model was performed to test within-person effects with free trait levels. In the second complementary analysis, we used the HI-ALT model as an alternative method to test the within-person effect, also taking into account the latent growth of the constructs over time [75]. In the third complementary analysis, we used the ALT-SR model, which is a combination of the RI-CLPM and the HI-ALT models, to model the latent growth and the structured residuals of the observed variables. The fourth complementary analysis was a fully saturated CLPM model [76], which controls for all lag-2 and lag-3 effects. Lastly, we performed the single-level random effects model [77], but obtained near zero variances of the random effects (all < 0.001); and the XM model [78], which obtained small and non-significant XM interaction effects. The specifications of the CLPM, RI-CLPM, and ALT-SR model followed the Mplus syntax of Orth et al. [44], while the HI-ALT model was specified as in Ou et al. [75]. The latent growth factors of cognitive function, depressive symptoms, and physical activity in the HI-ALT and ALT-SR models, and the observed variables in the CLPM and RI-CLPM models were regressed on sex, age, and sex × age. Bootstrapped 10,000 replications were estimated to obtain asymmetric confidence intervals.

## Data sharing

This SHARE dataset is available at http://www.share-project.org/data-access.html.

## Supplementary information

Supplemental Material
