# Supplementary material for: Physical activity partly mediates the association between cognitive function and depressive symptoms
Source: Transl Psychiatry. 2022 Sep 27;12:414. doi: 10.1038/s41398-022-02191-7 (PMC9515096; doi:10.1038/s41398-022-02191-7)
Supplement: Supplementary file 1 — Supplemental Material [file 41398_2022_2191_MOESM1_ESM.docx]

| **Supplementary Table 1.** Sensitivity and complementary analyses | | | | | | | | | | | | | | | | | | |
| --- | --- | --- | --- | --- | --- | --- | --- | --- | --- | --- | --- | --- | --- | --- | --- | --- | --- | --- |
|  | **AIC** | **X2(df)** | **RMSEA**  **(95% CI)** | **CFI** | **TLI** | **SRMR** | **a1** | **b1** | **c1’ (direct effect)** | **total effect 1** | **indirect effect 1** | **proportion of mediation effect 1** | **a2** | **b2** | **c2’ (direct effect)** | **total effect 2** | **indirect effect 2** | **proportion of mediation effect 1** |
| **Sensitivity analysis 1** (using vigorous instead of moderate physical activity; CLPM) | | | | | | | | | | | |  |  |  |  |  |  |  |
| Model 1 | 1615089.115 | 10229.083(37)*** | .073 (.072-.075) | .914 | .762 | .059 | .030*** | -.065*** | -.038*** | -.040*** | -.002*** | 5% | .037*** | -.075*** | -.042*** | -.045*** | -.003*** | 7% |
| Model 2 | 1615038.132 | 10178.100(37)*** | .073 (.072-.074) | .914 | .763 | .059 | .065*** | -.041*** | -.068*** | -.070*** | -.003*** | 4% | .081*** | -.051*** | -.091*** | -.095*** | -.004*** | 4% |
| **Sensitivity analysis 2** (using verbal fluency instead of delayed recall as the measure of cognitive function; CLPM) | | | | | | | | | | | |  |  |  |  |  |  |  |
| Model 1 | 1143820.062 | 7686.644(37)*** | .064 (.062-.065) | .923 | .788 | .058 | .087*** | -.132*** | -.130*** | -.142*** | -.011*** | 8% | .133*** | -.136*** | -.152*** | -.170*** | -.018*** | 11% |
| Model 2 | 1144440.781 | 8307.363(37)*** | .066 (.065-.067) | .917 | .771 | .063 | .034*** | -.128*** | -.111*** | -.115*** | -.004*** | 3% | .039*** | -.137*** | -.094*** | -.099*** | -.005*** | 5% |
| **Complementary analysis 1** (RI-CLPM) | | | | | | | | | | | |  |  |  |  |  |  |  |
| Model 1 | 1518574.217 | 454.199(31)*** | .016 (.015-.018) | .996 | .987 | .014 | -.014** | -.077*** | .004 | .005 | .001** | 20% | -.002 | -.086** | -.023 | -.022 | .000 | 0% |
| Model 2 | 1518620.908 | 500.889(31)*** | .017 (.016-.019) | .996 | .986 | .005 | -.024 | -.003 | -.022 | -.022 | .000 | 0% | .008 | -.016 | -.020 | -.020 | .000 | 0% |
| **Complementary analysis 2** (HI-ALT) | | | | | | | | | | | |  |  |  |  |  |  |  |
| Model 1 | 1518207.775 | 105.757(40)*** | .006 (.004-.007) | .999 | .998 | .006 | -.006* | -.005 | .021** | .021** | .000 | 0% | -.005 | .070** | .005 | .005 | .000 | 0% |
| Model 2 | 1518210.064 | 108.046(40)*** | .006 (.004-.007) | .999 | .998 | .008 | .000 | .018** | -.005 | -.005 | .000 | 0% | .001 | .011 | .061** | .061** | .000 | 0% |
| **Complementary analysis 3** (ALT-SR) | | | | | | | | | | | |  |  |  |  |  |  |  |
| Model 1 | 1518533.665 | 431.646(40)*** | .014 (.013-.015) | .996 | .991 | .010 | -.007 | -.022 | .005 | .005 | .000 | 0% | -.003 | .078* | .000 | .000 | .000 | 0% |
| Model 2 | 1518540.227 | 438.208(40)*** | .014 (.013-.015) | .996 | .991 | .010 | .026 | .008 | -.020 | -.020 | .000 | 0% | -.003 | .020 | .043 | .043 | .000 | 0% |
| **Complementary analysis 4** (Fully saturated CLPM; specific indirect effect reported only through the proposed mediator) | | | | | | | | | | | | |  |  |  |  |  |  |
| Model 1 | 1518182.018 | 0(0) | .000 (.000-.000) | 1.000 | 1.000 | .000 | .021*** | -.100*** | -.021*** | -.023*** | -.002*** | 4% | .016*** | -.079*** | -.027* | -.028* | -.001** | 2% |
| Model 2 |  |  |  |  |  |  | .100*** | -.025*** | -.084*** | -.087*** | -.003*** | 2% | .059*** | -.016 | -.037 | -.038 | -.001 | 1% |

Note. * p < .05. ** p < .01. *** p < .001. CLPM: cross-lagged panel longitudinal mediation model; RI-CLPM: random intercepts cross-lagged panel longitudinal mediation model; HI-ALT: history-independent autoregressive latent trajectory longitudinal mediation model; ALT-SR: autoregressive latent trajectory model with structured residuals and longitudinal mediation. AIC: Akaike Information Criterion. CFI: comparative fit index; CI: confidence interval; RMSEA: root mean square error of approximation; SRMR: standardized root mean squared residual; TLI: Tucker-Lewis index. The observed depressive symptoms, physical activity, and cognitive function variables, or the latent growth factors of depressive symptoms, physical activity, and cognitive function were regressed on sex, age, and sex × age in each wave. Model 1: cognitive function 🡪 (a) physical activity 🡪 (b) depressive symptoms; Model 2: physical activity 🡪 (a) cognitive function 🡪 (b) depressive symptoms.

**Mplus syntaxes:**

**Main analysis, Model 1:**

MISSING is blank;

USEVAR =

moder4 moder5 moder6 moder7

wordD4 wordD5 wordD6 wordD7

DEP4 DEP5 DEP6 DEP7

age sex sexage;

DEFINE:

sexage = sex*age;

ANALYSIS:

bootstrap = 10000;

MODEL:

moder4 moder5 moder6 moder7 on sex age sexage;

wordD4 wordD5 wordD6 wordD7 on sex age sexage;

DEP4 DEP5 DEP6 DEP7 on sex age sexage;

dep4 with wordD4;

dep4 with moder4;

wordD4 with moder4;

dep5 with wordD5;

dep5 with moder5;

wordD5 with moder5;

dep6 with wordD6;

dep6 with moder6;

wordD6 with moder6;

dep7 with wordD7;

dep7 with moder7;

wordD7 with moder7;

wordD5 on wordD4;

wordD6 on wordD5;

wordD7 on wordD6;

DEP5 on DEP4;

DEP6 on DEP5;

DEP7 on DEP6;

moder5 on moder4;

moder6 on moder5;

moder7 on moder6;

!mediation: wordD-moder-DEP

moder5 on wordD4;

dep6 on moder5;

dep6 on wordD4;

moder6 on wordD5;

dep7 on moder6;

dep7 on wordD5;

dep5 on moder4 ;

moder7 on wordD6;

Model indirect:

DEP6 ind wordD4;

DEP7 ind wordD5;

OUTPUT: SAMPSTAT STDYX RESIDUAL CINTERVAL(bootstrap);

**Main analysis, Model 2:**

MISSING is blank;

USEVAR =

moder4 moder5 moder6 moder7

wordD4 wordD5 wordD6 wordD7

DEP4 DEP5 DEP6 DEP7

sex age sexage;

DEFINE:

sexage = sex*age;

ANALYSIS:

bootstrap = 10000;

MODEL:

moder4 moder5 moder6 moder7 on sex age sexage;

wordD4 wordD5 wordD6 wordD7 on sex age sexage;

DEP4 DEP5 DEP6 DEP7 on sex age sexage;

dep4 with wordD4 ;

dep4 with moder4 ;

wordD4 with moder4 ;

dep5 with wordD5 ;

dep5 with moder5 ;

wordD5 with moder5 ;

dep6 with wordD6 ;

dep6 with moder6 ;

wordD6 with moder6 ;

dep7 with wordD7 ;

dep7 with moder7 ;

wordD7 with moder7 ;

wordD5 on wordD4 ;

wordD6 on wordD5 ;

wordD7 on wordD6 ;

DEP5 on DEP4 ;

DEP6 on DEP5 ;

DEP7 on DEP6 ;

moder5 on moder4 ;

moder6 on moder5 ;

moder7 on moder6 ;

!mediation: moder-wordD-DEP

wordD5 on moder4 ;

dep6 on wordD5 ;

dep6 on moder4 ;

wordD6 on moder5 ;

dep7 on wordD6 ;

dep7 on moder5 ;

wordD7 on moder6;

dep5 on wordD4;

Model indirect:

DEP6 ind moder4;

DEP7 ind moder5;

OUTPUT: SAMPSTAT STDYX RESIDUAL CINTERVAL(bootstrap);

**Complementary analysis 1, Model 1:**

MISSING is blank;

USEVAR =

moder4 moder5 moder6 moder7

wordD4 wordD5 wordD6 wordD7

DEP4 DEP5 DEP6 DEP7

age sex sexage;

DEFINE:

sexage = sex*age;

ANALYSIS:

MODEL = NOCOVARIANCES;

bootstrap = 10000;

MODEL:

moder4 moder5 moder6 moder7 on sex age sexage;

wordD4 wordD5 wordD6 wordD7 on sex age sexage;

DEP4 DEP5 DEP6 DEP7 on sex age sexage;

!random intercept factors

moder by moder4@1 moder5@1 moder6@1 moder7@1;

[moder@0]; moder*;

[moder4-moder7]; moder4-moder7@0;

wordD by wordD4@1 wordD5@1 wordD6@1 wordD7@1;

[wordD@0]; wordD*;

[wordD4-wordD7]; wordD4-wordD7@0;

dep by DEP4@1 DEP5@1 DEP6@1 DEP7@1;

[dep@0]; dep*;

[dep4-dep7]; dep4-dep7@0;

moder wordD dep with moder wordD dep;

!wave-specific residual factors

moderR4 by moder4@1;

moderR5 by moder5@1;

moderR6 by moder6@1;

moderR7 by moder7@1;

[moderR4-moderR7@0];

moderR4-moderR7;

wordDR4 by wordD4@1;

wordDR5 by wordD5@1;

wordDR6 by wordD6@1;

wordDR7 by wordD7@1;

[wordDR4-wordDR7@0];

wordDR4-wordDR7;

depR4 by dep4@1;

depR5 by dep5@1;

depR6 by dep6@1;

depR7 by dep7@1;

[depR4-depR7@0];

depR4-depR7;

!structural relations between residual factors

depR4 with wordDR4 ;

depR4 with moderR4 ;

wordDR4 with moderR4 ;

depR5 with wordDR5 ;

depR5 with moderR5 ;

wordDR5 with moderR5 ;

depR6 with wordDR6 ;

depR6 with moderR6 ;

wordDR6 with moderR6 ;

depR7 with wordDR7 ;

depR7 with moderR7 ;

wordDR7 with moderR7 ;

wordDR5 on wordDR4 ;

wordDR6 on wordDR5 ;

wordDR7 on wordDR6 ;

depR5 on depR4 ;

depR6 on depR5 ;

depR7 on depR6 ;

moderR5 on moderR4 ;

moderR6 on moderR5 ;

moderR7 on moderR6 ;

!mediation: wordD-moder-DEP

moderR5 on wordDR4 ;

depR6 on moderR5 ;

depR6 on wordDR4 ;

moderR6 on wordDR5 ;

depR7 on moderR6 ;

depR7 on wordDR5 ;

depR5 on moderR4 ;

moderR7 on wordDR6;

Model indirect:

depR6 ind wordDR4;

depR7 ind wordDR5;

OUTPUT: SAMPSTAT STDYX RESIDUAL CINTERVAL(bootstrap);

**Complementary analysis 1, Model 2:**

MISSING is blank;

USEVAR =

moder4 moder5 moder6 moder7

wordD4 wordD5 wordD6 wordD7

DEP4 DEP5 DEP6 DEP7

sex age sexage;

DEFINE:

sexage = sex*age;

ANALYSIS:

MODEL = NOCOVARIANCES;

bootstrap = 10000;

MODEL:

moder4 moder5 moder6 moder7 on sex age sexage;

wordD4 wordD5 wordD6 wordD7 on sex age sexage;

DEP4 DEP5 DEP6 DEP7 on sex age sexage;

!random intercept factors

moder by moder4@1 moder5@1 moder6@1 moder7@1;

[moder@0]; moder*;

[moder4-moder7]; moder4-moder7@0;

wordD by wordD4@1 wordD5@1 wordD6@1 wordD7@1;

[wordD@0]; wordD*;

[wordD4-wordD7]; wordD4-wordD7@0;

dep by DEP4@1 DEP5@1 DEP6@1 DEP7@1;

[dep@0]; dep*;

[dep4-dep7]; dep4-dep7@0;

moder wordD dep with moder wordD dep;

!wave-specific residual factors

moderR4 by moder4@1;

moderR5 by moder5@1;

moderR6 by moder6@1;

moderR7 by moder7@1;

[moderR4-moderR7@0];

moderR4-moderR7;

wordDR4 by wordD4@1;

wordDR5 by wordD5@1;

wordDR6 by wordD6@1;

wordDR7 by wordD7@1;

[wordDR4-wordDR7@0];

wordDR4-wordDR7;

depR4 by dep4@1;

depR5 by dep5@1;

depR6 by dep6@1;

depR7 by dep7@1;

[depR4-depR7@0];

depR4-depR7;

!structural relations between residual factors

depR4 with wordDR4 ;

depR4 with moderR4 ;

wordDR4 with moderR4 ;

depR5 with wordDR5 ;

depR5 with moderR5 ;

wordDR5 with moderR5 ;

depR6 with wordDR6 ;

depR6 with moderR6 ;

wordDR6 with moderR6 ;

depR7 with wordDR7 ;

depR7 with moderR7 ;

wordDR7 with moderR7 ;

wordDR5 on wordDR4 ;

wordDR6 on wordDR5 ;

wordDR7 on wordDR6 ;

depR5 on depR4 ;

depR6 on depR5 ;

depR7 on depR6 ;

moderR5 on moderR4 ;

moderR6 on moderR5 ;

moderR7 on moderR6 ;

!mediation: moder-wordD-DEP

wordDR5 on moderR4 ;

depR6 on wordDR5 ;

depR6 on moderR4 ;

wordDR6 on moderR5 ;

depR7 on wordDR6 ;

depR7 on moderR5 ;

wordDR7 on moderR6;

depR5 on wordDR4;

Model indirect:

DEPR6 ind moderR4;

DEPR7 ind moderR5;

OUTPUT: SAMPSTAT STDYX RESIDUAL CINTERVAL(bootstrap);

**Complementary analysis 2, Model 1:**

MISSING is blank;

USEVAR =

moder4 moder5 moder6 moder7

wordD4 wordD5 wordD6 wordD7

DEP4 DEP5 DEP6 DEP7

age sex sexage;

DEFINE:

sexage = sex*age;

ANALYSIS:

MODEL = NOCOVARIANCES;

bootstrap = 10000;

MODEL:

iD sD | DEP4@0 DEP5@1 DEP6@2 DEP7@3;

iC sC | wordD4@0 wordD5@1 wordD6@2 wordD7@3;

iP sP | moder4@0 moder5@1 moder6@2 moder7@3;

iD sD iC sC iP sP with iD sD iC sC iP sP;

iD sD iC sC iP sP on sex age sexage;

!structural relations between observed variables

dep4 with wordD4 ;

dep4 with moder4 ;

wordD4 with moder4 ;

dep5 with wordD5 ;

dep5 with moder5 ;

wordD5 with moder5 ;

dep6 with wordD6 ;

dep6 with moder6 ;

wordD6 with moder6 ;

dep7 with wordD7 ;

dep7 with moder7 ;

wordD7 with moder7 ;

wordD5 on wordD4 ;

wordD6 on wordD5 ;

wordD7 on wordD6 ;

DEP5 on DEP4 ;

DEP6 on DEP5 ;

DEP7 on DEP6 ;

moder5 on moder4 ;

moder6 on moder5 ;

moder7 on moder6 ;

!mediation: wordD-moder-DEP

moder5 on wordD4 ;

dep6 on moder5 ;

dep6 on wordD4 ;

moder6 on wordD5 ;

dep7 on moder6 ;

dep7 on wordD5 ;

dep5 on moder4 ;

moder7 on wordD6;

Model indirect:

DEP6 ind wordD4;

DEP7 ind wordD5;

OUTPUT: SAMPSTAT STDYX RESIDUAL CINTERVAL(bootstrap);

**Complementary analysis 2, Model 2:**

MISSING is blank;

USEVAR =

moder4 moder5 moder6 moder7

wordD4 wordD5 wordD6 wordD7

DEP4 DEP5 DEP6 DEP7

sex age sexage;

DEFINE:

sexage = sex*age;

ANALYSIS:

MODEL = NOCOVARIANCES;

bootstrap = 10000;

MODEL:

iD sD | DEP4@0 DEP5@1 DEP6@2 DEP7@3;

iC sC | wordD4@0 wordD5@1 wordD6@2 wordD7@3;

iP sP | moder4@0 moder5@1 moder6@2 moder7@3;

iD sD iC sC iP sP with iD sD iC sC iP sP ;

iD sD iC sC iP sP on sex age sexage;

!structural relations between observed variables

dep4 with wordD4 ;

dep4 with moder4 ;

wordD4 with moder4 ;

dep5 with wordD5 ;

dep5 with moder5 ;

wordD5 with moder5 ;

dep6 with wordD6 ;

dep6 with moder6 ;

wordD6 with moder6 ;

dep7 with wordD7 ;

dep7 with moder7 ;

wordD7 with moder7 ;

wordD5 on wordD4 ;

wordD6 on wordD5 ;

wordD7 on wordD6 ;

DEP5 on DEP4 ;

DEP6 on DEP5 ;

DEP7 on DEP6 ;

moder5 on moder4 ;

moder6 on moder5 ;

moder7 on moder6 ;

!mediation: moder-wordD-DEP

wordD5 on moder4 ;

dep6 on wordD5 ;

dep6 on moder4 ;

wordD6 on moder5 ;

dep7 on wordD6 ;

dep7 on moder5 ;

wordD7 on moder6;

dep5 on wordD4;

Model indirect:

DEP6 ind moder4;

DEP7 ind moder5;

OUTPUT: SAMPSTAT STDYX RESIDUAL CINTERVAL(bootstrap);

**Complementary analysis 3, Model 1:**

MISSING is blank;

USEVAR =

moder4 moder5 moder6 moder7

wordD4 wordD5 wordD6 wordD7

DEP4 DEP5 DEP6 DEP7

age sex sexage;

DEFINE:

sexage = sex*age;

ANALYSIS:

MODEL = NOCOVARIANCES;

bootstrap = 10000;

MODEL:

iD sD | DEP4@0 DEP5@1 DEP6@2 DEP7@3;

iC sC | wordD4@0 wordD5@1 wordD6@2 wordD7@3;

iP sP | moder4@0 moder5@1 moder6@2 moder7@3;

[iD*];iD*;

[sD*];sD*;

[DEP4-DEP7@0];DEP4-DEP7@0;

[iC*];iC*;

[sC*];sC*;

[wordD4-wordD7@0];wordD4-wordD7@0;

[iP*];iP*;

[sP*];sP*;

[moder4-moder7@0];moder4-moder7@0;

iD sD iC sC iP sP with iD sD iC sC iP sP ;

iD sD iC sC iP sP on sex age sexage;

!wave-specific residual factors

moderR4 by moder4@1;

moderR5 by moder5@1;

moderR6 by moder6@1;

moderR7 by moder7@1;

[moderR4-moderR7@0];

moderR4-moderR7;

wordDR4 by wordD4@1;

wordDR5 by wordD5@1;

wordDR6 by wordD6@1;

wordDR7 by wordD7@1;

[wordDR4-wordDR7@0];

wordDR4-wordDR7;

depR4 by dep4@1;

depR5 by dep5@1;

depR6 by dep6@1;

depR7 by dep7@1;

[depR4-depR7@0];

depR4-depR7;

!structural relations between residual factors

depR4 with wordDR4 ;

depR4 with moderR4 ;

wordDR4 with moderR4 ;

depR5 with wordDR5 ;

depR5 with moderR5 ;

wordDR5 with moderR5 ;

depR6 with wordDR6 ;

depR6 with moderR6 ;

wordDR6 with moderR6 ;

depR7 with wordDR7 ;

depR7 with moderR7 ;

wordDR7 with moderR7 ;

wordDR5 on wordDR4 ;

wordDR6 on wordDR5 ;

wordDR7 on wordDR6 ;

depR5 on depR4 ;

depR6 on depR5 ;

depR7 on depR6 ;

moderR5 on moderR4 ;

moderR6 on moderR5 ;

moderR7 on moderR6 ;

!mediation: wordD-moder-DEP

moderR5 on wordDR4 ;

depR6 on moderR5 ;

depR6 on wordDR4 ;

moderR6 on wordDR5 ;

depR7 on moderR6 ;

depR7 on wordDR5 ;

depR5 on moderR4 ;

moderR7 on wordDR6;

Model indirect:

depR6 ind wordDR4;

depR7 ind wordDR5;

OUTPUT: SAMPSTAT STDYX RESIDUAL CINTERVAL(bootstrap);

**Complementary analysis 3, Model 2:**

MISSING is blank;

USEVAR =

moder4 moder5 moder6 moder7

wordD4 wordD5 wordD6 wordD7

DEP4 DEP5 DEP6 DEP7

sex age sexage;

DEFINE:

sexage = sex*age;

ANALYSIS:

MODEL = NOCOVARIANCES;

bootstrap = 10000;

MODEL:

iD sD | DEP4@0 DEP5@1 DEP6@2 DEP7@3;

iC sC | wordD4@0 wordD5@1 wordD6@2 wordD7@3;

iP sP | moder4@0 moder5@1 moder6@2 moder7@3;

[iD*];iD*;

[sD*];sD*;

[DEP4-DEP7@0];DEP4-DEP7@0;

[iC*];iC*;

[sC*];sC*;

[wordD4-wordD7@0];wordD4-wordD7@0;

[iP*];iP*;

[sP*];sP*;

[moder4-moder7@0];moder4-moder7@0;

iD sD iC sC iP sP with iD sD iC sC iP sP ;

iD sD iC sC iP sP on sex age sexage;

!wave-specific residual factors

moderR4 by moder4@1;

moderR5 by moder5@1;

moderR6 by moder6@1;

moderR7 by moder7@1;

[moderR4-moderR7@0];

moderR4-moderR7;

wordDR4 by wordD4@1;

wordDR5 by wordD5@1;

wordDR6 by wordD6@1;

wordDR7 by wordD7@1;

[wordDR4-wordDR7@0];

wordDR4-wordDR7;

depR4 by dep4@1;

depR5 by dep5@1;

depR6 by dep6@1;

depR7 by dep7@1;

[depR4-depR7@0];

depR4-depR7;

!structural relations between residual factors

depR4 with wordDR4 ;

depR4 with moderR4 ;

wordDR4 with moderR4 ;

depR5 with wordDR5 ;

depR5 with moderR5 ;

wordDR5 with moderR5 ;

depR6 with wordDR6 ;

depR6 with moderR6 ;

wordDR6 with moderR6 ;

depR7 with wordDR7 ;

depR7 with moderR7 ;

wordDR7 with moderR7 ;

wordDR5 on wordDR4 ;

wordDR6 on wordDR5 ;

wordDR7 on wordDR6 ;

depR5 on depR4 ;

depR6 on depR5 ;

depR7 on depR6 ;

moderR5 on moderR4 ;

moderR6 on moderR5 ;

moderR7 on moderR6 ;

!mediation: moder-wordD-DEP

wordDR5 on moderR4 ;

depR6 on wordDR5 ;

depR6 on moderR4 ;

wordDR6 on moderR5 ;

depR7 on wordDR6 ;

depR7 on moderR5 ;

wordDR7 on moderR6;

depR5 on wordDR4;

Model indirect:

DEPR6 ind moderR4;

DEPR7 ind moderR5;

OUTPUT: SAMPSTAT STDYX RESIDUAL CINTERVAL(bootstrap);

**Complementary analysis 4:**

MISSING is blank;

USEVAR =

moder4 moder5 moder6 moder7

wordD4 wordD5 wordD6 wordD7

DEP4 DEP5 DEP6 DEP7

age sex sexage;

DEFINE:

sexage = sex*age;

ANALYSIS:

bootstrap = 10000;

MODEL:

moder4 moder5 moder6 moder7 on sex age sexage;

wordD4 wordD5 wordD6 wordD7 on sex age sexage;

DEP4 DEP5 DEP6 DEP7 on sex age sexage;

dep4 with wordD4;

dep4 with moder4;

wordD4 with moder4;

dep5 with wordD5;

dep5 with moder5;

wordD5 with moder5;

dep6 with wordD6;

dep6 with moder6;

wordD6 with moder6;

dep7 with wordD7;

dep7 with moder7;

wordD7 with moder7;

wordD5 on wordD4;

wordD6 on wordD5 wordD4;

wordD7 on wordD6 wordD5 wordD4;

DEP5 on DEP4;

DEP6 on DEP5 DEP4;

DEP7 on DEP6 DEP5 DEP4;

moder5 on moder4;

moder6 on moder5 moder4;

moder7 on moder6 moder5 moder4;

wordD5 on DEP4;

wordD6 on DEP5 DEP4;

wordD7 on DEP6 DEP5 DEP4;

wordD5 on moder4;

wordD6 on moder5 moder4;

wordD7 on moder6 moder5 moder4;

DEP5 on wordD4;

DEP6 on wordD5 wordD4;

DEP7 on wordD6 wordD5 wordD4;

DEP5 on moder4;

DEP6 on moder5 moder4;

DEP7 on moder6 moder5 moder4;

moder5 on wordD4;

moder6 on wordD5 wordD4;

moder7 on wordD6 wordD5 wordD4;

moder5 on DEP4;

moder6 on DEP5 DEP4;

moder7 on DEP6 DEP5 DEP4;

Model indirect:

DEP6 ind wordD4;

DEP7 ind wordD5;

DEP6 ind moder4;

DEP7 ind moder5;

wordD6 ind DEP4;

wordD7 ind DEP5;

wordD6 ind moder4;

wordD7 ind moder5;

moder6 ind DEP4;

moder7 ind DEP5;

moder6 ind wordD4;

moder7 ind wordD5;

OUTPUT: SAMPSTAT STDYX RESIDUAL CINTERVAL(bootstrap);
